# Spatial scaling of pollen-plant diversity relationship in landscapes with contrasting diversity patterns

**DOI:** 10.1038/s41598-022-22353-3

**Published:** 2022-10-26

**Authors:** Vojtěch Abraham, Petr Kuneš, Ondřej Vild, Eva Jamrichová, Zuzana Plesková, Barbora Werchan, Helena Svitavská-Svobodová, Jan Roleček

**Affiliations:** 1 Department of Botany, Faculty of Science, Charles University, Benátská 2, 12800 Praha, Czech Republic; 2 Department of Paleoecology, Institute of Botany of the Czech Academy of Sciences, Lidická 25/27, 602 00 Brno, Czech Republic; 3 Department of Botany and Zoology, Faculty of Science, Masaryk University, Kotlářská 2, 611 37 Brno, Czech Republic; 4 Department of Vegetation Ecology, Institute of Botany of the Czech Academy of Sciences, Lidická 25/27, 602 00 Brno, Czech Republic; 5 German Pollen Information Service Foundation, Charitéplatz 1, 10117 Berlin, Germany

**Keywords:** Palaeoecology, Biodiversity

## Abstract

Mitigating the effects of global change on biodiversity requires its understanding in the past. The main proxy of plant diversity, fossil pollen record, has a complex relationship to surrounding vegetation and unknown spatial scale. We explored both using modern pollen spectra in species-rich and species-poor regions in temperate Central Europe. We also considered the biasing effects of the trees by using sites in forests and open habitats in each region. Pollen samples were collected from moss polsters at 60 sites and plant species were recorded along two 1 km-transects at each site. We found a significant positive correlation between pollen and plant richness (alpha diversity) in both complete datasets and for both subsets from open habitats. Pollen richness in forest datasets is not significantly related to floristic data due to canopy interception of pollen rather than to pollen productivity. Variances (beta diversity) of the six pollen and floristic datasets are strongly correlated. The source area of pollen richness is determined by the number of species appearing with increasing distance, which aggregates information on diversity of individual patches within the landscape mosaic and on their compositional similarity. Our results validate pollen as a reconstruction tool for plant diversity in the past.

## Introduction

Quantification of the ongoing biodiversity changes calls upon improving our knowledge of past biodiversity and its dynamics during the Cenozoic. The study of this period allows analysis of plant diversity using the most common palaeoecological proxy, i.e. pollen record^1^. In addition, during this period took place the most similar warming event to the present global change, the Palaeocene-Eocene boundary, when rising temperature increased diversity^2^. The warming after the end of the Pleistocene meant a decrease in diversity due to the spread of temperate forest^3^ and human impact raised it again by disturbances^4,5^. For the last seven thousand years, man counts as a relevant factor, which, moreover, altered the ecological rules^4,6^. Past diversity of plants needs to be studied within the context of all possible factors and for this purpose, knowledge of the spatial scaling of the pollen-plant diversity relationship is essential.

Available comparisons of modern pollen richness and plant species richness in the surrounding vegetation have mostly revealed a positive relationship between the two variables^7–13^, and only rarely are opposite results obtained^14^. However, the floristic data used in these studies came from non-uniform sources and covered different spatial scales. The local scale^7–12^, corresponding to alpha diversity as conceived by ecological studies, was usually captured by field surveys of the surrounding vegetation. Field surveys require extensive plant searching and good identification skills and are therefore time and personnel intensive; the areas surveyed are thus relatively small. Only two studies^8,9^ measured the spatial scale of plant richness relevant for pollen richness, but it still remains largely unknown. Available broad-scale studies^13,14^ have relied on floristic data stored in databases and floras, which often suffer from low spatial resolution and variable taxonomic detail^15^. As a consequence, the resulting plant richness estimates corresponded more to gamma diversity as conceived in ecological studies. We suggest that cross-scale studies based on plant diversity data sampled at high spatial resolution over broader areas are therefore needed.

Pollen richness estimates may also be biased by the presence of species with high pollen production, such as *Pinus* or *Betula*^16,17^. These taxa tend to dominate the pollen rain and decrease the probability of detecting rare taxa that are often represented by one or a few pollen grains. Applying representation factors^18^ or pollen productivity estimates helps to balance the representation of different taxa and can lead to a stronger positive relationship between pollen- and plant richness^13^. However, this correction was possible only in areas with available values of pollen productivity estimates, all in northern Europe. One part of the present dataset was previously used for calculation of pollen productivity estimates^19^, and so it provides a good opportunity to test this effect in the temperate zone.

Another important aspect of plant diversity whose history we should understand better is beta diversity^20^. Beta diversity can be thought of as compositional turnover across space or time, and several attempts have been made to estimate beta diversity from the fossil pollen record using various indices of beta diversity^21^. Directional metrics can be estimated between pairs of pollen communities using dissimilarity coefficients, e.g. rate-of-change analysis^22,23^; or within a set of communities using, e.g. the length of the gradient in ordination space^24^. Perhaps only a single study has found a positive relationship between pollen- and vegetation turnover calculated from data in a 60 × 60 km grid^25^. Other calibration experiments have approximated beta diversity by different measures of landscape structure^26,27^ and therefore we anticipate that studies treating forest and open habitats separately may provide a deeper understanding of the relationship between pollen- and plant beta diversity.

**Figure 1:**
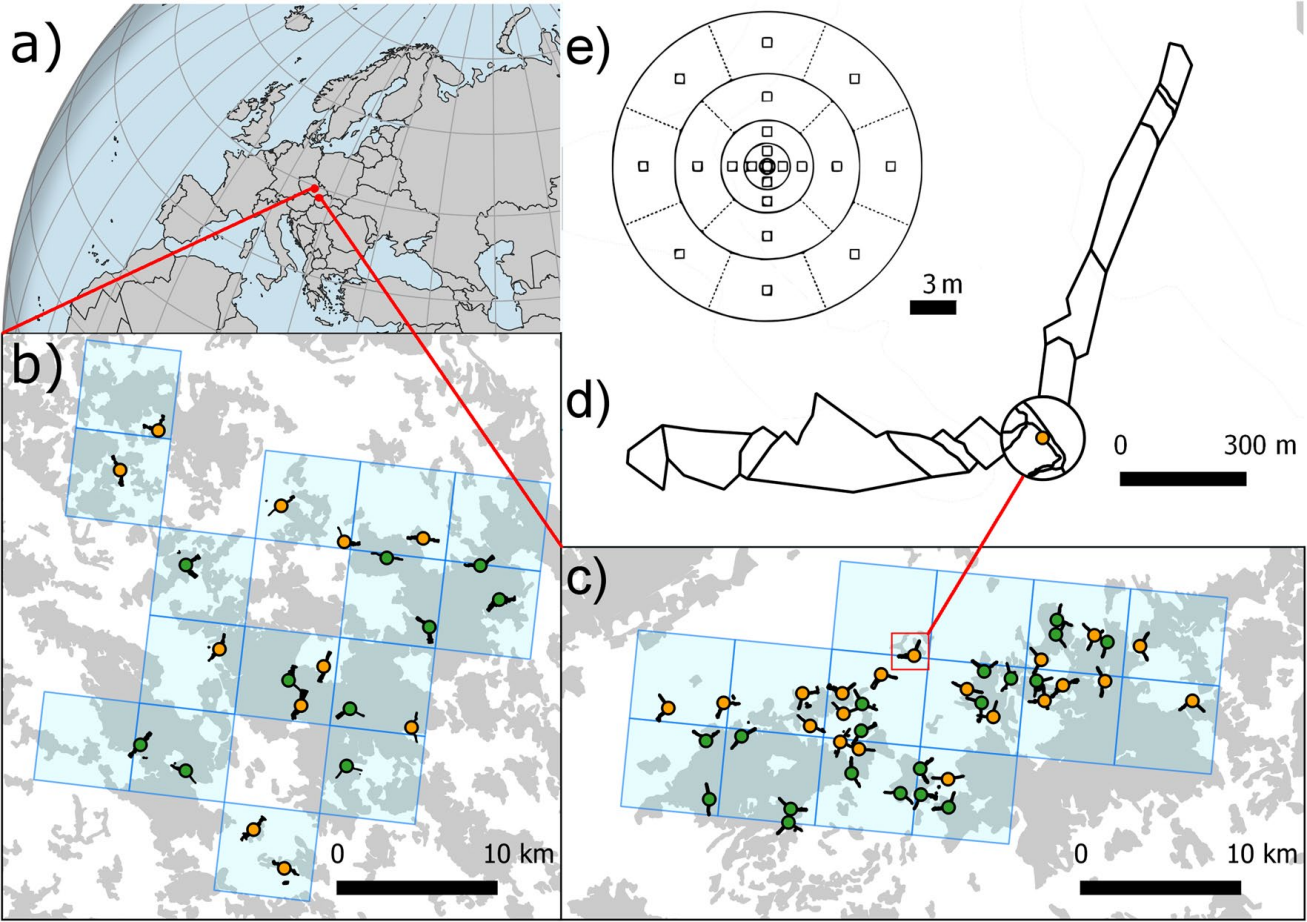
Map of the study areas showing (**a**) position within Europe, (**b**) BMH: Bohemian-Moravian Highlands, (**c**) WCM: White Carpathians. Yellow and green circles indicate sites in open habitats and forest, respectively. Blue squares show the area of the reference plant diversity data from the PLADIAS database. Grey indicates forested area. Short lines represent transects of the vegetation survey, (**d**) circle 10–100 m and two transects of polygons recording the plant diversity 100–1000 m, (**e**) 21 plots within 0–10 m.

In this study, we investigated the strength of the relationship between pollen- and plant diversity. We focused on forested and open habitats in species-rich (Fig. 1c) and species-poor regions (Fig. 1b) of temperate Central Europe (Fig. 1a), in order to obtain and compare datasets with contrasting patterns of plant diversity and with different dominant vegetation as a potential biasing factor of the pollen record through high pollen production of trees. Furthermore, we sampled plant diversity data with high spatial resolution over a relatively large area, which allowed us to assess the effect of distance for which plant diversity is considered. Finally, in addition to alpha diversity (richness), we assessed the relationships between beta diversities (variances) of pollen- and plant composition data.

## Results

### Patterns of richness and beta diversity

**Figure 2:**
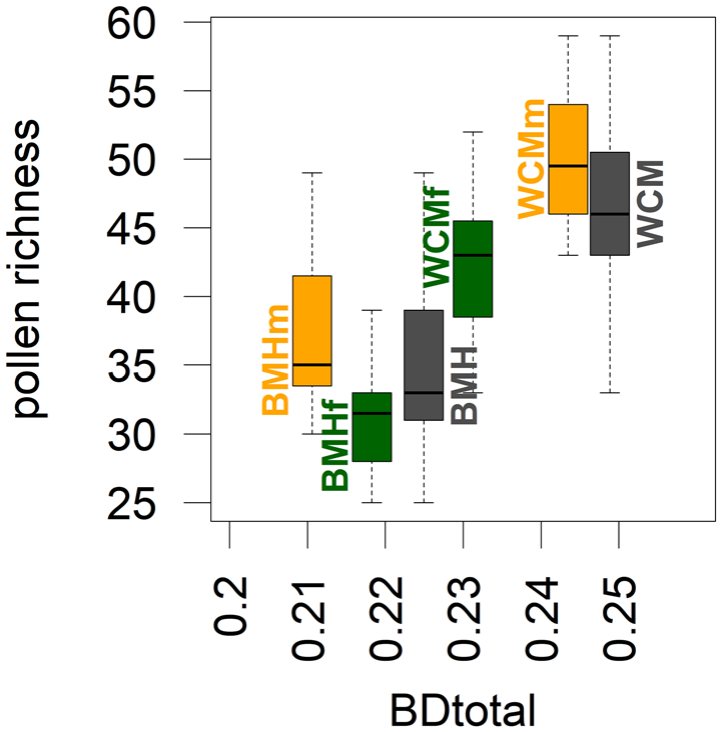
Pollen alpha diversity (pollen richness, y-axis) and beta diversity (BD_Total_, x-axis) in two study regions and their different habitats. Open habitats (yellow), forest (green), and both habitats together (black).

**Figure 3:**
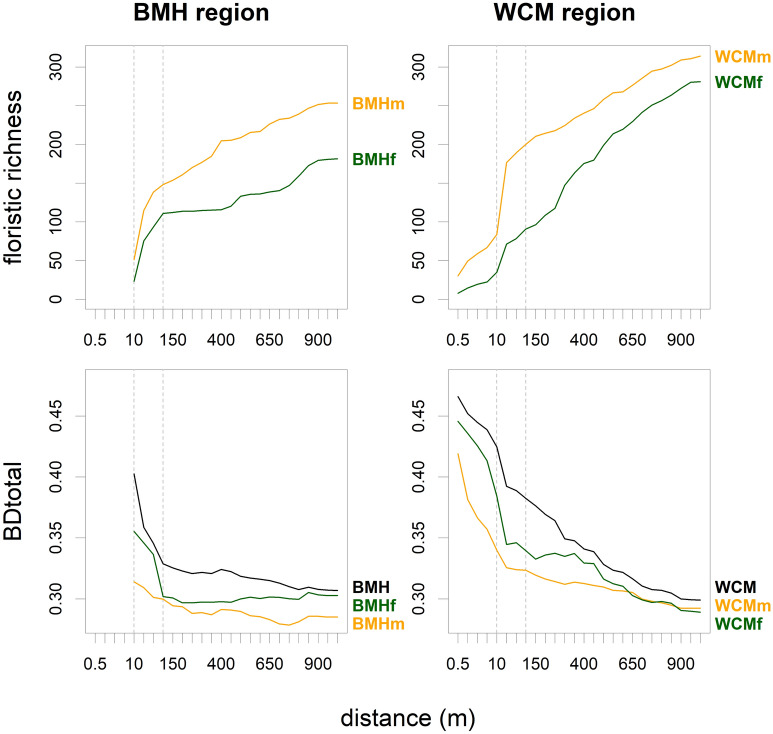
Spatial scaling of plant alpha diversity (species richness) and beta diversity (BD_Total_) in two study regions and their different habitats. The mean number of plant species appearing in the vegetation survey (top) and their total variance (bottom). Open habitats are indicated by the yellow line, forest by the green line, and both habitats together by the black line.

In total, we found 169 pollen types (95 in the Bohemian-Moravian Highlands, hereafter BMH, and 151 in the White Carpathian Mts., hereafter WCM) and 1323 plant species (799 in BMH and 1098 in WCM). Mean pollen richness per sample ranged from 30.9 in the BMH forest subset, 38.0 in the BMH open-habitat subset, 42.0 in the WCM forest subset, to 50.1 in the WCM open-habitat subset (Fig. 2). Plant richness had the same rank order for the radius between 600 and 1000 m. In the WCM forest subset, the increase with increasing radius was more or less gradual, whereas in the other subsets it was initially steeper, with more than half of the species appearing in the first 100 m (Fig. 3). The open-habitat subsets in both regions gained most species within the meadow-forest mosaics surrounding the central point. The richness of the BMH forest subset came largely from man-made habitats (forest roads at 10 to 100 m and built-up areas usually at distances over 500 m), whereas the WCM forest subset was enriched with species from grasslands and other semi-natural habitats, usually at distances over 200 m (Fig. 5).

Beta-diversity (measured by BD_Total_) in the complete datasets was always higher than in their subsets, both for pollen and plants. BD_Total_ values in WCM were higher than in BMH up to 700 m for both pollen and plants. BD_Total_ values in the pollen subsets ranged from 0.21 in the BMH open-habitat subset to 0.24 in the WCM open-habitat subset (Fig. 2). BD_Total_ of plant composition data was highest in the WCM forest subset and lowest in the BMH open-habitat subset considering the distance between 40 and 200 m. The general decreasing trend with increasing distance showed only minor exceptions, the most conspicuous being the increase at 150 m in the WCM forest subset (Fig. 3).

### Pollen-plant diversity relationship

**Figure 4:**
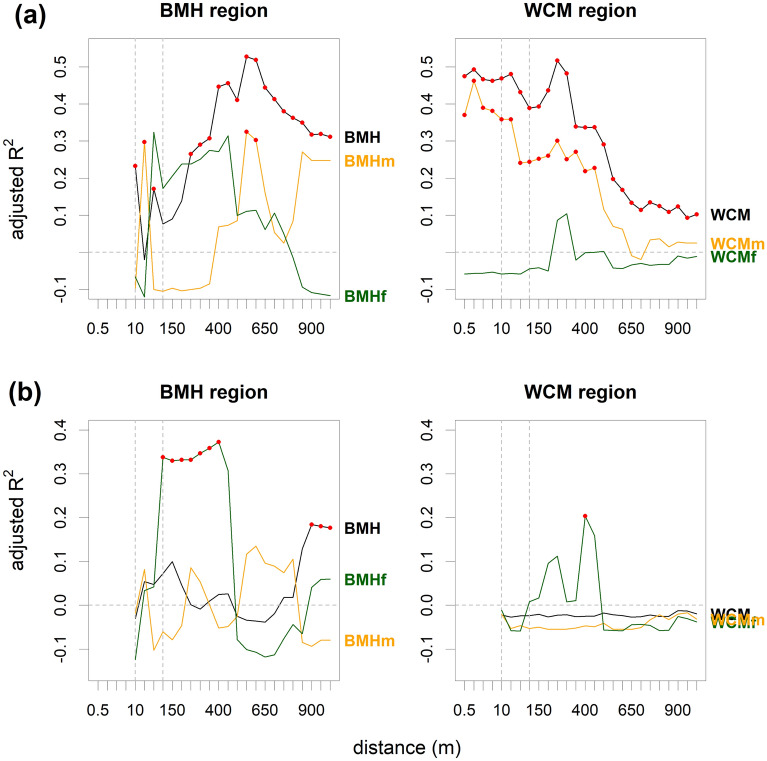
The strength of linear regression between (**a**) pollen richness and plant richness at different distances from sampling sites and (**b**) local contributions of sites to pollen and plant BD_Total_ at different distances from sampling sites. The black line shows the correlation for all sites, the orange line for sites in open habitats, and the green line for forest sites. Red dots indicate significant correlations.

All datasets showed a positive correlation between pollen and plant richness for at least some distances (Fig. 4a). Both complete datasets and the WCM open-habitat subset showed a highly significant correlation, while both BMH subsets showed a less significant correlation (but see lower sample size in the latter). WCM forest subset showed only a marginally significant correlation, despite a higher sample size (Supplementary Table S3 and Fig. S1). The adjusted R^2^ for pollen- and plant richness generally showed two distance ranges where the correlation was high.

**Figure 5:**
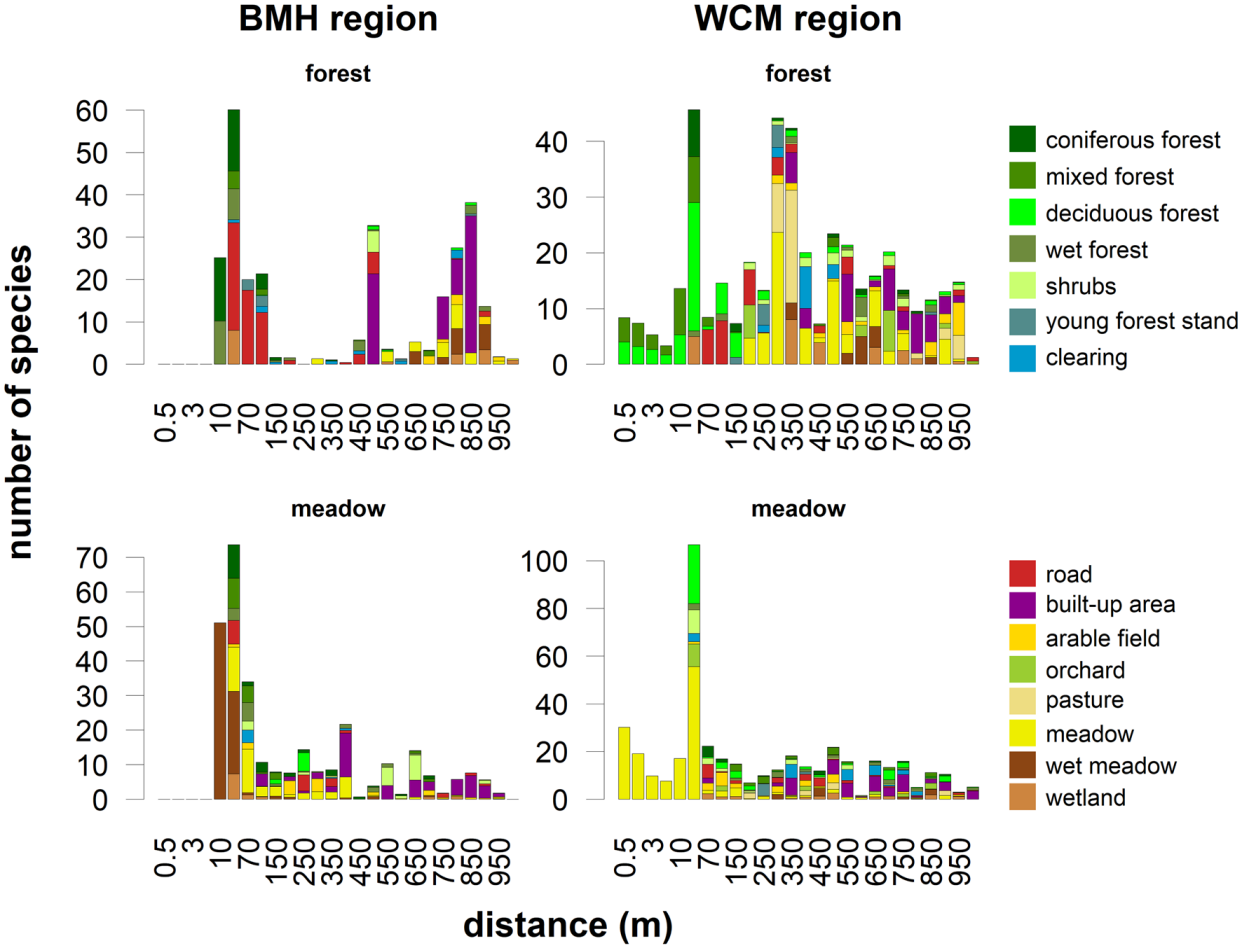
Numbers of new species recorded with increasing radius in different study regions and habitats. Colours code habitats, where the plants species were recorded.

The first range of distances fall within a few tens of meters from the central point (1.5–70 m) in all datasets except the WCM forest (Fig. 4a). At this small distance, most species are appearing for the first time (Fig. 5). The highest correlation of pollen- and plant richness in the WCM open-habitat subset is at a distance of 1.5 m within the steppic meadows with extremely high fine-scale species richness. The BMH open-habitat subset showed a local maximum of adjusted R^2^ at 40 m where new habitats such as forests frequently appear. The BMH forest subset showed the best match at 70 m, where open-habitat species growing along forest roads often appear (Fig. 5).

The second range of maximum adjusted R^2^ values occurred between 400 and 550 m in the BMH open-habitat subset and between 250 and 300 m in the WCM forest subset. These distances correspond to the appearance of a high number of new species in built-up areas and grasslands, respectively. The local maximum in adjusted R^2^ in the BMH forest subset at 450 m correlated with low numbers of species (< 10) growing along forest roads, clearings and wet forests; in contrast, high numbers of species (> 30) appearing around 500 m were accompanied by a decrease in adjusted R^2^.

The pollen assemblages from the forest habitats are dominated by tree pollen; from the open-sites by herb pollen (Supplementary Fig. S2), however in the forest we also identified higher abundance of herb pollen from species, which ecologically belong to open habitats (Supplementary Fig. S3). The adjustment of pollen counts by productivity estimates lead to lower or the same adjusted R^2^ values in all datasets. The only exception is subset from open-habitats in WCM, where both pollen richness values adjusted by pollen productivity estimates show stronger correlation with floristic richness (Supplementary Fig. S4).

**Figure 6:**
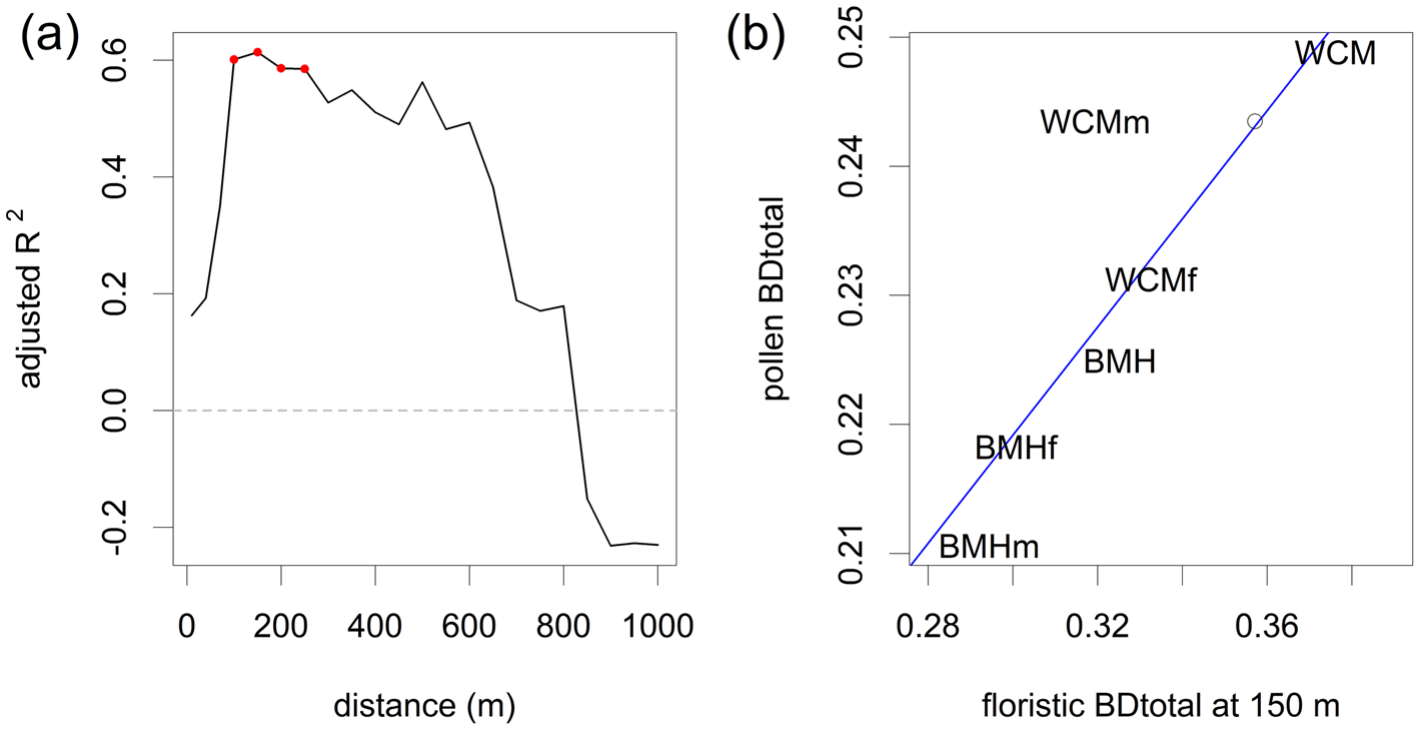
Relationship between pollen and floristic beta diversity (BD_Total_) (**a**) for the distance of 150 m. Empty dots indicate plant beta diversity (BD_Total_) at 6 m in WCM open-habitat-subset. (**b**) Adjusted R^2^ at different distances from sampling sites. Red dots indicate significant correlations.

The highest adjusted R^2^ between pollen and plant BD_Total_ was found at a distance of 150 m. Significant correlations occurred between 100 and 250 m, and a remarkably high, albeit non-significant, correlation occurred between 300 and 600 m (Fig. 6a). The plant BD_Total_ at 150 m was lower in the WCM open-habitat subset than in the other datasets, standing out from the otherwise linear relationship with the pollen BD_Total_ (Fig. 6b).

Local contributions of sites to pollen- and plant BD_Total_ values were significantly correlated in both forest subsets between 100 and 400 m and in the complete BMH dataset between 900 and 1000 m. The BMH open-habitat subset showed a positive but non-significant relationship, while complete dataset and open-habitat subset from the WCM region dataset showed no relationship (Fig. 4b).

## Discussion

We found a significant positive relationship between pollen- and plant richness regardless of differences in plant diversity, landscape structure and environmental conditions between the two study regions. This finding represents a major step stone towards more accurate paleoecological reconstructions of plant diversity in temperate Central Europe, as previous studies on this topic have mostly been conducted in boreal and boreal-nemoral zones^8,11^, in high mountain habitats^10^ or in southern Europe^9,12^.

Methodological differences e.g., in diversity indices, data transformations or sample sizes used make comparison between studies difficult. Nevertheless, the strongest relationships seem to be found when habitats with contrasting patterns of plant diversity are compared, such as forests and alpine vegetation^7^ or forests, peatlands and grasslands^11^. Also in our study, we found the strongest correlations when complete datasets combining forested and open habitats were analysed together for both study regions. As it is well known that plant richness is generally lower in forests than in open landscapes across temperate and boreal regions^28^, this finding may seem rather trivial. However, it is important for paleoecological reconstruction because Holocene changes in diversity in temperate regions were largely driven by changes in the relative abundance of major habitat types (such as forests, grasslands, wetlands and man-made habitats), and not just by changes in species richness within these habitats^5,6^.

Regarding individual habitats, the pollen-plant diversity relationship is often rather strong and significant in grasslands and other open habitats^8,11^; for example the WCM open-habitat subset in this study. Open habitats are generally richer in species, thus providing a longer gradient of species richness compensating for the taxonomical imprecision of the pollen analysis. In forested sites with less species, we found mostly non-significant relationships. Moreover, two other factors may play a role.

First, high pollen productivity of trees biases the diversity relationship according to the studies from northern Europe^16^. However, a study from an elevational transect in southern Norway showed that the strongest bias in representation occurs only in the boreal forest biome, which is dominated by high pollen producers^10^. Our dominant vegetation component, *Picea* and *Quercus*, have intermediate to high pollen productivity (2–2.5), whereas true high pollen producers such as *Alnus* and *Betula* (> 3) are less abundant in our study area (Supplementary Fig. S2). Adjustment of pollen counts by PPEs led to stronger relationship between pollen and floristic richness only in the WCM open-habitat subset (Supplementary Fig. S4).

Second, interception of pollen by the tree canopies^29^ and subsequent washout to the forest floor affects the diversity relationship of forest sites more than pollen productivity. This noise described also as a vegetation filtering^30^ can be illustrated in our dataset by pollen of long-distance transport from *Ambrosia artemisiifolia*-type, which has the closest source populations ca. 50 km south-eastwards from WCM region^31^; or pollen of *Artemisia*, growing in open habitats. Both pollen taxa are more abundant in the forest than in open sites (Supplementary Fig. S3).

Regarding the application of these results for the interpretation of fossil record, we suggest to consider only marked changes of pollen richness in the past and to avoid overinterpretation of small differences, as the non-significant relationships obtained in both forest datasets suggest some limitations of the method.

We showed that the pollen-plant diversity relationship may be at least partly disentangled by knowing the exact spatial position of plant species in broader surroundings of the pollen sampling sites. Changes in the relationship with changing spatial scale are largely driven by the numbers of species newly appearing as the radius of surveyed area increases, especially as new habitats are added (Fig. 5, Supplementary Fig. S5). Remarkably, in the BMH region it increases with distance, whereas the opposite trend was observed in the WCM region. This discrepancy may be explained by non-uniform richness patterns in different habitats and by different landscape structure (i.e. spatial arrangement of different habitats) in the two study regions.

At open-habitat sites in the WCM area, most species generally appeared within the first 40 m. This observation is consistent with the knowledge of extremely high fine-scale plant diversity in the local steppic meadows, where a substantial portion of the species pool occurs on a scale of tens of square meters^32^. Moreover, the grain size of the habitat mosaic in the WCM region is finer than in the BMH region. Therefore, the closest pollen-plant diversity relationship across habitats in the WCM region is achieved over shorter distances. Although habitats such as built-up areas and roads occurring at distances greater than 40 m may be species-rich and compositionally different from the grasslands and forests, it appears that high fine-scale plant diversity (in our case in WCM open-habitat subset) limits the influence of the surrounding landscape on pollen richness and reduces the source area of pollen richness. Several studies of the relevant source area of pollen report analogous results^33–35^. A weakening relationship between pollen diversity and plant diversity with distance has also been observed in the Mediterranean region^9^, although their interpretations are limited by field survey methodology.

The appearance of open habitats within forests led to the increase of species numbers and the local maxima of adjusted R^2^ in both regions. While in the BMH forest the appearance of forest roads at about 70 m was crucial, meadows and orchards at about 250 m played a similar role in the WCM forest subset. In the WCM open-habitat subset diversity patterns in the first tens of metres were crucial, while in the BMH open-habitat subset increased correlation of floristic and pollen richness appeared only at 400 and 550 m; at this distance many species appeared due to the frequent transition of meadow complexes to shrubby habitats and built-up areas. Also other studies from semi-open landscapes found a high correlation between pollen richness and landscape openness^17,26,27^.

Estimating the source area of pollen variance as a regression of pollen and floristic variance implies that the resulting distance of 100–250 m represents all datasets. Although they differ in species richness, openness and habitats, the relationship between variances is fairly linear. The exception is the WCM open-habitat subset suggesting that the spatial scale at which the pollen variance corresponds to the floristic variance cannot be generalized.

The strong effect of high pollen richness in the WCM open-habitat subset is also visible in the comparison of pollen and floristic variance. At 150 m, the WCM open-habitat subset had much lower floristic variance than the other subsets. Floristic variance in this subset corresponding to the pollen variance and the pattern of the other datasets lay at 6 m (Fig. 6b). Again, this may be caused by the high fine-scale diversity of the meadows, which include most pollen types present in the surrounding landscape. Only a few new species appeared in broader surroundings and at 150 m, WCM open habitats are more similar than other analysed habitats. The fact that extremely high alpha diversity is compensated by low beta diversity has already been reported from the open habitats of the White Carpathians^36^. The linearity and the significance of the variance relationship within the rest of the datasets indicate robustness and possible applicability to a variety of fossil records.

The mechanism of establishing the source area of pollen variance was similar to that mentioned for the source area of pollen richness. The appearance of new habitats with new species (Fig. 5) like open habitat for forest sites (WCM forest subset) or built-up areas for open sites (BMH open-habitat subset), caused small to negligible increases of floristic variance. Moreover, the high yet insignificant relationship of the variances at the distance between 250 and 600 m (Fig. 6a) corresponds to the distance of the second range of fit between floristic and pollen richness (Fig. 4a).

Beta diversity, understood as directional turnover (temporal or spatial), is becoming more frequently used in pollen analysis^22,24^ than beta diversity as a non-directional variation. According to Nieto-Lugilde et al.^25^ pollen-based turnover correlates with forest-inventory-based turnover. We extend this finding from woody taxa to all species and from directional turnover to non-directional variance. Moreover, forest sites with high contributions to pollen beta diversity also show an increased contribution to floristic beta diversity (Fig. 4b).

The reference data on plant diversity report 1477 species in 15 mapping squares covered by our survey for the BMH region and 2045 species in 14 squares for the WCM region^37^. It means that we recorded 54.1 and 53.7%, respectively, of the known regional species pool in the two regions. We consider this as a rather good result and the close agreement in representativeness between the two regions speaks for consistency in data quality between the datasets. We advise that future studies covering wider areas and various biomes should preferentially use high-quality floristic data collected in targeted field surveys rather than database data or data from simplified field surveys. Only then we will be able to understand the pollen-plant diversity relationships more realistically and in a spatially explicit manner.

In order to interpret fossil pollen richness in the light of our present results, we need to consider landscape openness, which can be roughly inferred from the ratio of arboreal and non-arboreal pollen. Variation of pollen richness during the forest phases of the records should be interpreted more carefully, especially in cases of low variation. In all other cases, the pollen richness is significantly linked to the plant richness within a distance of ten to several hundreds of meters, depending on the distance of the expected species-rich patches.

## Methods

### Study area

Our two study areas were situated in the temperate zone of Central Europe (Fig. 1). They represent semi-open landscapes with forests dominated mostly by spruce or oak (Kuneš et al.^19^). The two landscapes also differ in habitat conditions and plant diversity patterns. The model area for the low-diversity region was the highest part of the Bohemian-Moravian Highlands, called the Žďárské vrchy Mts. The Bohemian-Moravian Highlands (hereafter BMH) is the largest upland in the Czech Republic and its bedrock is mainly acid crystalline rocks. The landscape is mostly covered with plantations of *Picea abies*, with patches of low-productive meadows, wetlands and agricultural fields concentrated around the villages. The area is relatively poor in plant species and it is assumed that forests dominated its Holocene development^38^. The sampling sites (n = 21) were distributed over an area of 650 km^2^ at elevations between 570 and 760 m a.s.l.

The model area for the high-diversity region was the southwestern White Carpathians. The White Carpathian Mts (hereafter WCM) are located on the margin of the forest-steppe region of the Pannonian Basin^39,40^. Its bedrock is mainly base-rich sediments of the Carpathian flysch. The gently undulating landscape is covered by a varied mosaic of vegetation, including deciduous forests with a predominance of oak (*Quercus robur*), hornbeam (*Carpinus betulus*) and beech (*Fagus sylvatica*), as well as mown semi-natural steppic and mesic meadows, fields, orchards and vineyards. The area is considered a hotspot of fine-scale plant species richness^41,42^ and harbours a number of rare species with disjunct ranges^43^. Paleoecological studies indicate a long-term continuity of open habitats in this area^44^. It is part of the White Carpathians Protected Landscape Area and Biosphere Reserve. The sampling sites (n = 39) were distributed over an area of 250 km^2^ at elevations between 205 and 685 m a.s.l.

### Data sampling

In the low-diversity region (BMH), we sampled 10 sites in forested habitats and 11 sites in open habitats (wet meadows). In the high-diversity region (WCM), we sampled 19 sites in forested habitats and 20 sites in open habitats (steppic and mesic meadows). Forested sites were located in a continuous forested area, in a forest gap of at least 1 m^2^ to reduce the gravity component of pollen fallout not contributing to wind dispersal^45^. Open sites were selected in continuous non-forested habitats, with a minimum distance of at least 10 m from a mature tree.

Pollen samples were collected from a moss polster of at least 50 cm^2^ at the centre of each site. Plant composition data were collected within a 1 km radius (3.14 km^2^) around a central point in the same year (Supplementary Table S1). The vegetation sampling effort was divided into three zones: (i) complete species lists were recorded in the first 10 m (21 additional 1 m^2^ plots were sampled in the WCM to assess finer-scale relationships following a modified CRACKLES protocol^46^); (ii) between 10 and 100 m, the main vegetation types were mapped in the field using aerial photographs; the occurrence of additional species (not recorded in the first 10 m) was recorded for all mapped polygons; (iii) up to 1000 m, we recorded additional species along two 20 m wide linear transects; transect directions were selected based on aerial photographs to cover the greatest available habitat diversity; both transects had to have a minimum angular distance of 90°; within 1000 m, additional habitats not recorded along the transects were mapped and all additional species within them were recorded. In general, cultivated plants including ornamental plants (e.g., *Thuja*, *Bergenia*) were also recorded. We tried to avoid overlapping sampling sites while keeping the sampled area compact and homogeneous in terms of environmental conditions and vegetation types.

To check data completeness of our survey, we compared it with available data on species numbers in the national floristic database PLADIAS^37^. We considered all squares intersecting our transects.

Based on the data collected, we compiled six datasets: two ‘complete datasets’ including forested and open sites for both regions, and two ‘subsets’ with separate forested and open sites for each region.

### Statement

We obtained permissions from the Administrations of both Protected Landscape Areas (Ždárské Vrchy and Bílé Karpaty) to enter the study area and for collection of any plant and soil material, thus we fully complied with the local authorities and the national legislation. The vegetation survey was recorded in the field by non-lethal data collection. For the pollen survey, we picked small amount of moss (< 100 ml for each sample). Their scientific names are partly listed in Table S1, but none of them belongs to Species with Risk of Extinction. So, we fully complied also with the IUCN Policy Statement on Research Involving Species at Risk of Extinction and the Convention on the Trade in Endangered Species of Wild Fauna and Flora.

### Pollen analysis

Moss polsters were prepared for pollen analysis using standard procedures^47^. Moss samples were shaken in KOH overnight and then acetolyzed for 2 min. The pollen concentrate was stored in glycerine or silicone oil. Pollen slides were counted under a light microscope at 400 × magnification; for selected taxa at 1000 × magnification. The original pollen sum includes all pollen and spores of vascular plants according to the identification key of Beug^48^.

### Data analysis

Due to the varying pollen sum in the samples (from 943 to about 4000 grains), we reduced the sum to 943 grains per sample using random selection without replacement and repeated this procedure 100 times. The median number of taxa across repeated selections was used for further calculations.

In order to control the effect of taxa with high pollen productivity on the detection probability of less abundant pollen types and pollen richness of the samples, we adjusted pollen counts by pollen productivity estimates. The original pollen counts were divided by their pollen productivity estimate. Those values were calculated from the same pollen data in WCM and vegetation of regional scale. We considered the set of pollen productivity estimates with 25 taxa from the original study (Supplementary Table S2; Kuneš et al.^19^). Finally, we resampled the datasets to 520 grains per sample.

Pollen richness (number of pollen taxa) was then regressed against plant richness. Although the radius ranged from 0.5 to 1000 m, we considered pollen and plant richness at this scale to be alpha diversity. The concept of beta diversity in ecology is less equivocal and there are many definitions and alternative ways to calculate beta diversity^49^. Here, we use the total variance of the site-by-species community table as a measure of beta diversity^50^. The total variance represented by BD_Total_ value is a sum of squares in the site-by-species community table. We used Jaccard index on presence-absence data as its measure. The relative nature of BD_Total_, ranging from 0 to 1, allows us to compare different numbers of sites, and thus also to compare complete datasets and subsets thereof. Pollen BD_Total_ values calculated for our six datasets were regressed against plant BD_Total_ values calculated for six different radii around the sampling sites.

The calculation of beta diversity in the ‘adespatial’ package^50,51^ also allowed us to quantify the local contribution of each site to beta diversity (hereafter also referred to as ‘local contribution’) and its significance. We examined the relationship between pollen and floristic counterparts at different distances from the sampling sites, again using linear regression. The strength of the relationship was measured using an adjusted R^2^ for all diversity indicators examined (richness—alpha and gamma diversity; BD_Total_—beta diversity; local contribution). The source area of pollen diversity (pollen richness and pollen variance) was taken at the distance with the highest adjusted R^2^. The R software environment (version 3.4.3) was used for all statistical analyses^52^.

## Supplementary Information

Supplementary Information.

## Data Availability

Pollen data are available in the Neotoma Palaeoecological database. The list of the Neotoma datasets, vegetation data and further data at 10.5281/zenodo.7233824.

## Code availability

Code to reproduce the numerical analysis is available at https://github.com/vojtechabraham/SpatialScalingPollenDiversity/.
