# Supplementary material for: Spatial scaling of pollen-plant diversity relationship in landscapes with contrasting diversity patterns
Source: Sci Rep. 2022 Oct 26;12:17937. doi: 10.1038/s41598-022-22353-3 (PMC9606126; doi:10.1038/s41598-022-22353-3)
Supplement: Supplementary file 1 — Supplementary Information. [file 41598_2022_22353_MOESM1_ESM.pdf]

Supplementary Material to: Spatial scaling of pollen-plant diversity relationship in landscapes with contrasting diversity patterns

Vojtěch Abraham, Petr Kuneš, Ondřej Vild, Eva Jamrichová, Zuzana Plesková, Barbora Werchan, Helena Svitavská-Svobodová, Jan Roleček

Table S1. Sites.

reg	id	name	type	sampl. date	longitude	latitude	altitude	pollen sample material
BMH	3	Plíčky	open	29-MAR-2016	15.97328	49.56648	600 m	*Calliergonella cuspidata*
BMH	4	Louky u Černého lesa	open	29-MAR-2016	15.94290	49.58620	581 m	mosses
BMH	6	Račín	forest	29-MAR-2016	15.87842	49.61474	665 m	*Sphagnum* sect. *Squarrosum*
BMH	7	Vepřová-Žlábek	forest	29-MAR-2016	15.83664	49.62626	656 m	*Sphagnum* cf. *Girgensohnii*
BMH	10	Suché Kopce	open	29-MAR-2016	15.89515	49.68509	661 m	*Sphagnum* sect. *palustria*
BMH	11	Pihoviny	open	30-MAR-2016	15.97139	49.65886	676 m	*Sphagnum teres*
BMH	12	Kocanda	open	30-MAR-2016	15.98694	49.68227	644 m	Sphagnum sect. *palustria*
BMH	13	Porostliny	open	30-MAR-2016	16.06020	49.76057	590 m	*Sphagnum* cf. *Angustifolium*
BMH	14	Bahna	open	30-MAR-2016	15.99222	49.75349	655 m	*Sphagnum* sect. *palustria*
BMH	15	Ratajské rybníky	open	30-MAR-2016	15.93400	49.76956	592 m	*Sphagnum* sect. cf. *Cuspidata*
BMH	16	Zubří	open	30-MAR-2016	15.79089	49.77894	624 m	*Sphagnum* cf. *teres*
BMH	17	Nový Rybník	open	30-MAR-2016	15.81989	49.80366	572 m	*Sphagnum teres* + *S.* Sect. *Subsecunda*
BMH	18	Stropnická cesta	forest	30-MAR-2016	16.11238	49.74904	727 m	*Sphagnum* cf. *Girgensohnii*
BMH	19	Žižkov	forest	30-MAR-2016	16.13217	49.73117	760 m	*Sphagnum* cf. *Girgensohnii*
BMH	20	Samotín	open	30-MAR-2016	16.06899	49.65377	706 m	*Sphagnum* cf. *Fallax*
BMH	21	Chlum	forest	31-MAR-2016	15.85758	49.73012	569 m	*Sphagnum* cf. *Girgensohnii*
BMH	22	Míšek	forest	31-MAR-2016	16.03064	49.74724	683 m	*Sphagnum* cf. *Girgensohnii*
BMH	23	Knížecí studánka	forest	31-MAR-2016	16.07440	49.71121	724 m	*Sphagnum* sect. *palustria*
BMH	24	Pod Šindelným vrchem	forest	31-MAR-2016	15.95803	49.67219	739 m	*Sphagnum* cf. *Girgensohnii*
BMH	25	Rampoltův mlýn	forest	31-MAR-2016	16.01390	49.66028	704 m	*Sphagnum* cf. *Riparium*
BMH	26	Brožova skála	forest		16.01675	49.62796		mosses
WCM	2	B1	open	19-MAY-2013	17.53333	48.88450		mosses
WCM	4	B10	open	27-JUN-2013	17.52458	48.83287		mosses
WCM	6	B11	open	09-JUL-2013	17.55803	48.87033		mosses
WCM	8	B12	open	10-JUL-2013	17.43011	48.86400		mosses
WCM	10	B13	open	11-JUL-2013	17.42975	48.84800		mosses
WCM	12	B14	open	12-JUL-2013	17.40272	48.85531		mosses
WCM	14	B15	open	19-JUL-2013	17.44619	48.84497		mosses
WCM	36	B16	open	21-MAY-2014	17.28043	48.85752		mosses
WCM	38	B17	open	19-JUN-2013	17.48550	48.90047		mosses
WCM	39	B18	open	05-JUN-2014	17.39347	48.87321		mosses
WCM	40	B19	open	04-JUN-2013	17.42745	48.87539		mosses
WCM	20	B2	open	20-MAY-2013	17.59433	48.90500		mosses
WCM	41	B20	open	03-JUN-2013	17.45899	48.88813		mosses
WCM	23	B3	open	21-MAY-2013	17.60039	48.88214		mosses
WCM	25	B4	open	21-MAY-2013	17.61483	48.89183		mosses
WCM	27	B5	open	22-MAY-2013	17.65044	48.89639		mosses
WCM	29	B6	open	23-MAY-2013	17.67744	48.91792		mosses
WCM	31	B7	open	23-MAY-2013	17.72650	48.88992		mosses
WCM	33	B8	open	23-MAY-2013	17.63767	48.92156		mosses
WCM	35	B9	open	26-JUN-2013	17.32711	48.86350		mosses
WCM	17	L1	forest	18-JUN-2012	17.60442	48.92819		mosses
WCM	24	L10	forest	04-AUG-2012	17.44664	48.85539		mosses
WCM	32	L11	forest	05-AUG-2012	17.34517	48.84572		mosses
WCM	34	L12	forest	06-AUG-2012	17.31531	48.84117		mosses
WCM	3	L13	forest	06-AUG-2012	17.32297	48.80853		mosses
WCM	5	L14	forest	06-AUG-2012	17.52731	48.81708		mosses
WCM	11	L16	forest	07-AUG-2012	17.48517	48.82239		mosses
WCM	16	L17	forest	07-AUG-2012	17.39308	48.80753		mosses
WCM	13	L18	forest	29-JUL-2013	17.44197	48.83097		mosses
WCM	15	L19	forest	06-AUG-2013	17.39228	48.79989		mosses
WCM	18	L2	forest	20-JUN-2012	17.64928	48.91847		mosses
WCM	21	L20	forest	20-MAY-2014	17.60472	48.92004		mosses
WCM	1	L3	forest	21-JUN-2012	17.49997	48.83731		mosses
WCM	28	L4	forest	21-JUN-2012	17.54692	48.87761		mosses

WCM	30	L5	forest	23-JUL-2012	17.56997	48.89289	mosses
WCM	9	L6	forest	24-JUL-2012	17.50275	48.82275	mosses
WCM	26	L7	forest	25-JUL-2012	17.59197	48.89292	mosses
WCM	19	L8	forest	03-AUG-2012	17.54653	48.89542	mosses
WCM	22	L9	forest	04-AUG-2012	17.44522	48.87050	mosses

Table S2. Pollen productivity estimates (Kuneš et al 2019)

taxon	ppe25	ppe15
Abies	1.29	1.29
Acer	0.64	
Alnus	3.17	3.17
Matricaria-Typ	0.43	
Cichoroideae	0.58	
Betula pubescens type	3.37	3.37
Carpinus betulus	1.28	1.28
Corylus	3.28	3.28
Cyperaceae	1.88	1.88
Fagus	0.81	
Fraxinus excelsior-Typ	0.52	
Chenopodiaceae	1.5	1.5
Picea	2.17	2.17
Pinus sylvestris-Typ	1.61	1.61
Plantago lanceolata-Typ	2.88	2.88
Wildgras-Typ	1	1
Quercus	2.07	2.07
Ranunculus acris type*	0.96	
Rubiaceae	0.5	
Rumex acetosa-Typ	2.26	2.26
Salix	0.93	
Senecio/Aster	0.17	
Tilia	3.26	3.26
Ulmus	2.95	2.95
Urticaceae, Moraceae	0.51	

*) excl. *Pulsatilla, Anemone, R. aquatilis*

Table S3. Relationship of floristic and pollen richness within the datasets.

**region**	**dataset**	**n sites**	**distance max fit (m)**	**adjusted R^2**	**p_value**
BMH	forest	10	70	0.3235547	0.050
BMH	open	11	550	0.3201134	0.041*
BMH	all	21	550	0.525054	0.000***
WCM	forest	19	300	0.1037916	0.097
WCM	open	20	1.5	0.4623916	0.001***
WCM	All	39	250	0.5167158	0.000***

significance levels: ***<0.001, *<0.05

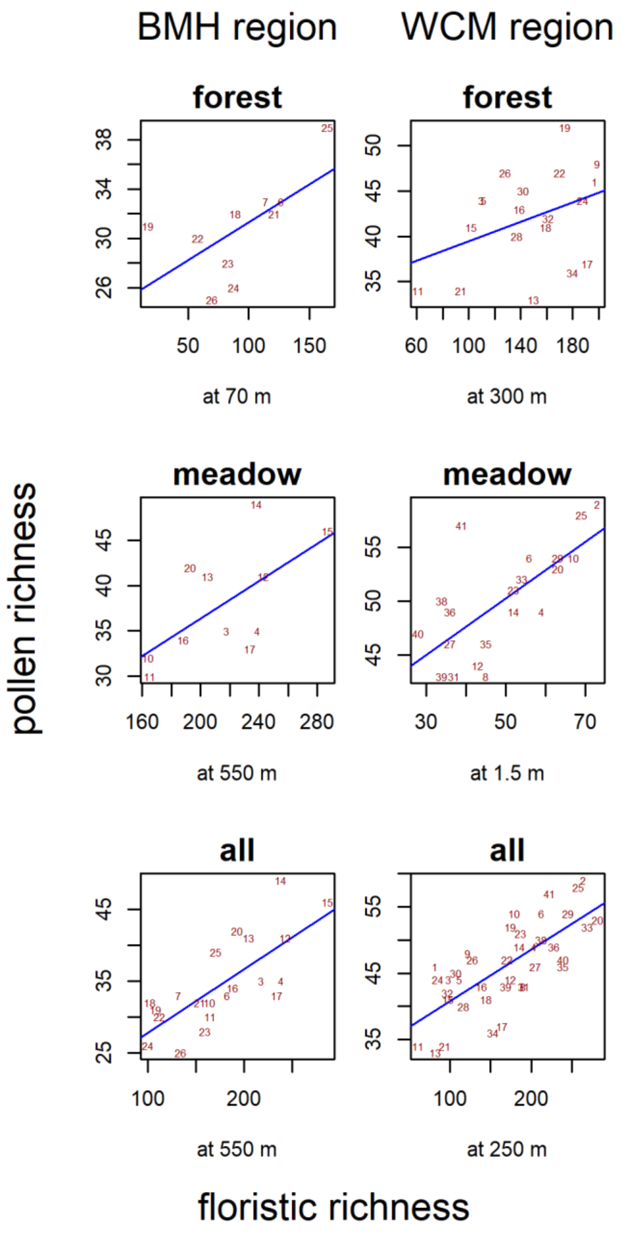

Fig. S1. The relationship between pollen richness and floristic richness at the distances with maximum overall correlation (see Table S3). Red numbers are sites' IDs (see Table S1).

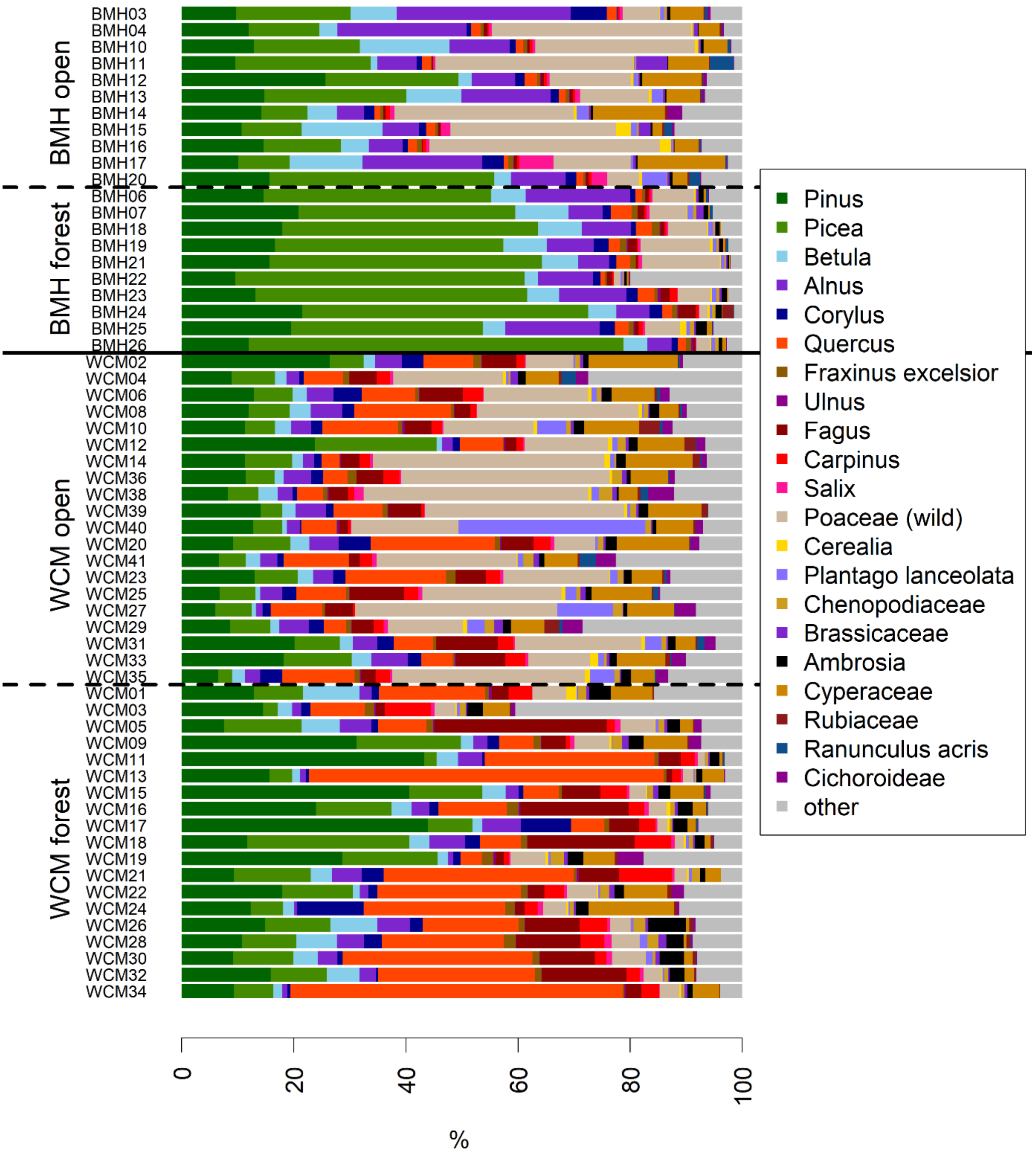

Fig. S2. Percentage composition of pollen assemblages.

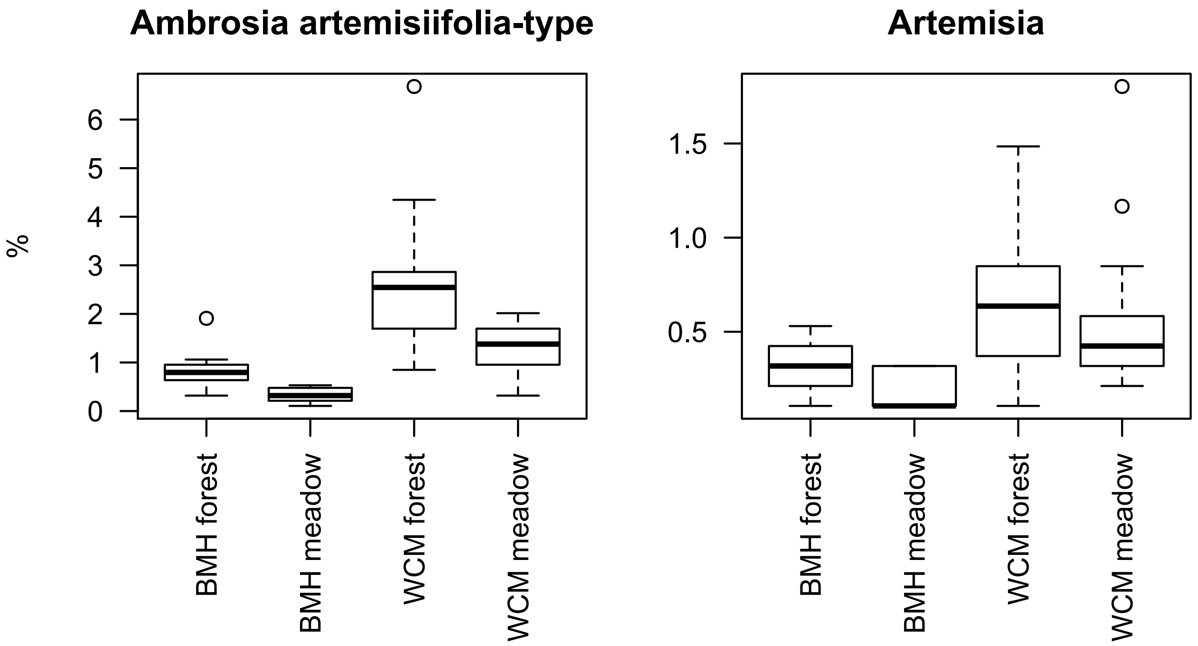

Fig. S3. Pollen percentages of herb taxa from open habitats which were more abundant in the forest.

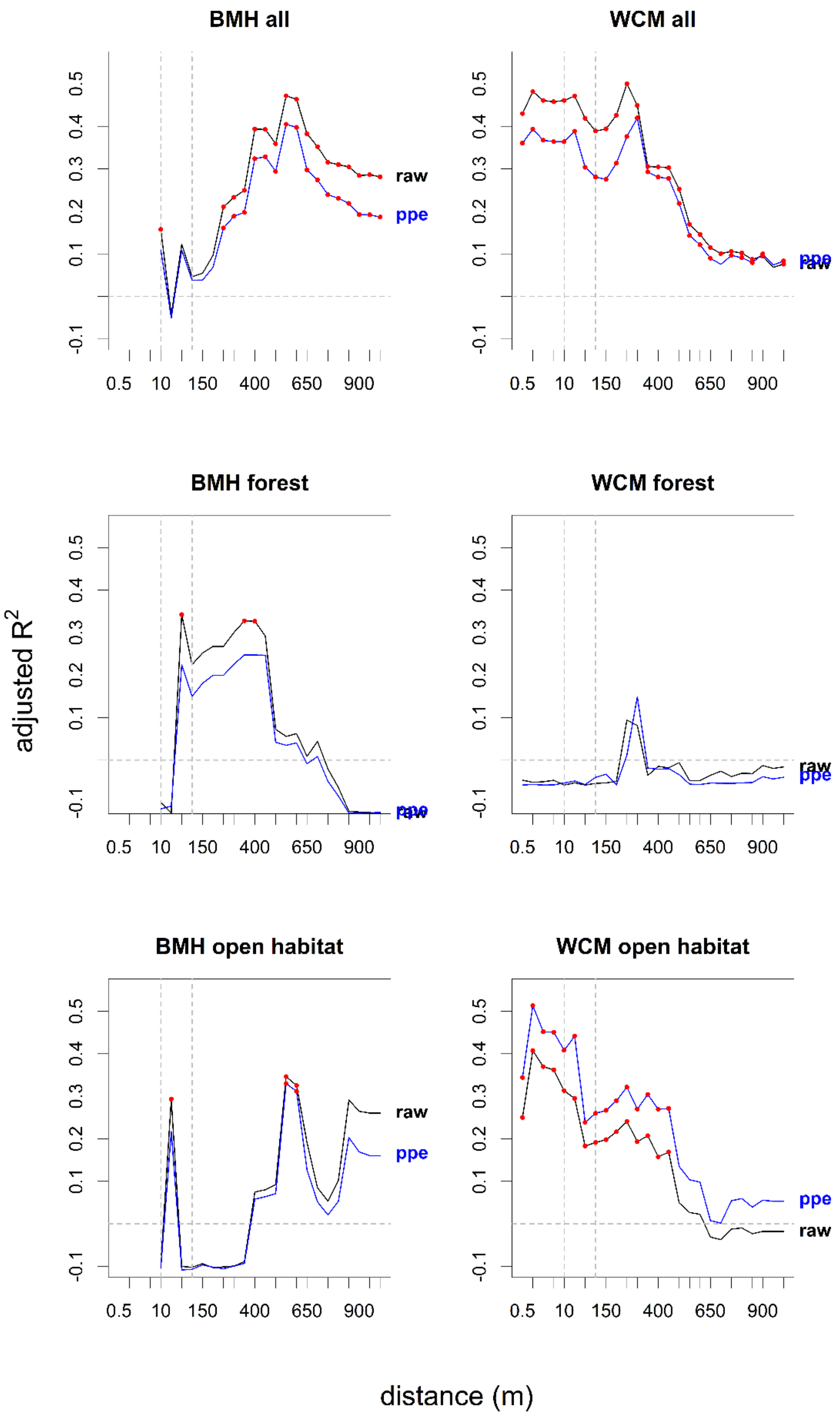

Fig. S4. The strength of linear regression between pollen richness based on original and ppe-adjusted pollen counts and plant richness at different distances from sampling sites

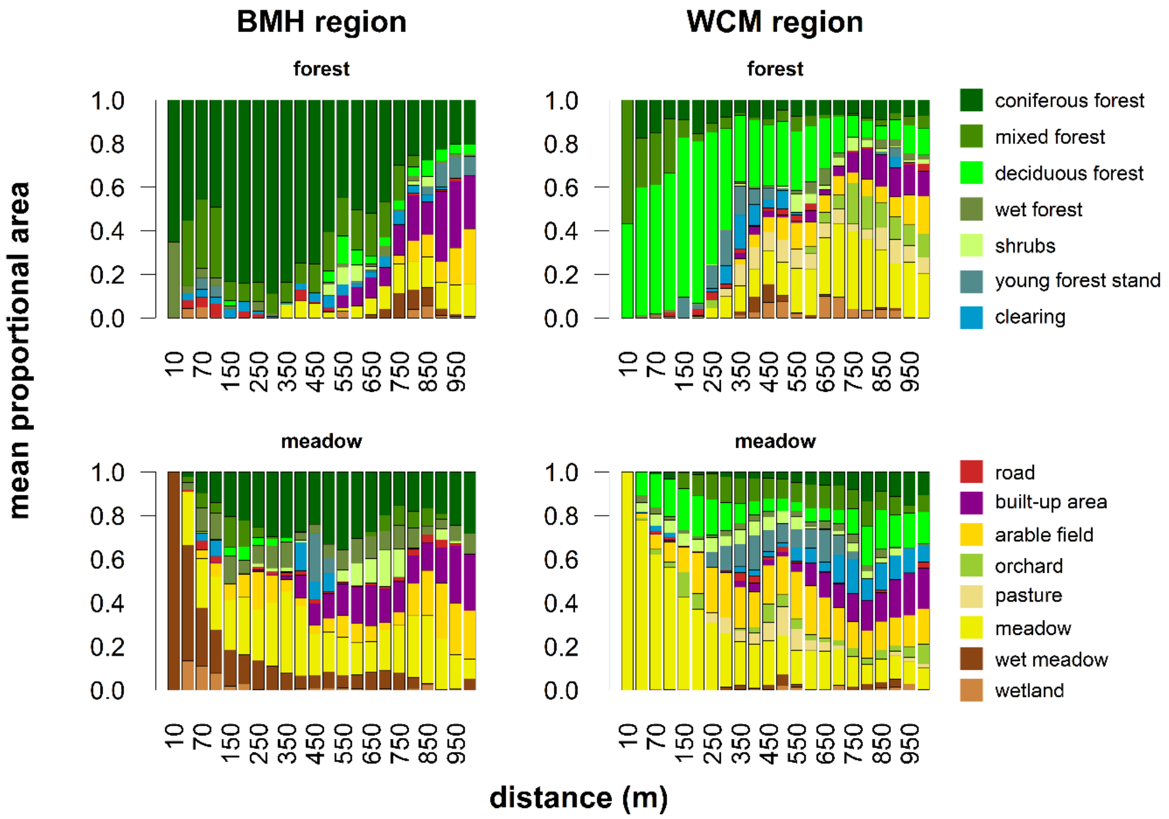

Fig. S5. Cumulative composition of habitats in the rings of the uniform datasets. The proportion of habitats corresponds to the relative area of each polygon within the ring.
